# Ancient Ancestry of KFDV and AHFV Revealed by Complete Genome Analyses of Viruses Isolated from Ticks and Mammalian Hosts

**DOI:** 10.1371/journal.pntd.0001352

**Published:** 2011-10-04

**Authors:** Kimberly A. Dodd, Brian H. Bird, Marina L. Khristova, César G. Albariño, Serena A. Carroll, James A. Comer, Bobbie R. Erickson, Pierre E. Rollin, Stuart T. Nichol

**Affiliations:** 1 Viral Special Pathogens Branch, Division of High Consequence Pathogens and Pathology, Centers for Disease Control and Prevention, Atlanta, Georgia, United States of America; 2 School of Veterinary Medicine, University of California Davis, Davis, California, United States of America; 3 Biotechnology Core Facility Branch, Division of Scientific Resources, Centers for Disease Control and Prevention, Atlanta, Georgia, United States of America; Texas Biomedical Research Institute, United States of America

## Abstract

**Background:**

Alkhurma hemorrhagic fever virus (AHFV) and Kyasanur forest disease virus (KFDV) cause significant human disease and mortality in Saudi Arabia and India, respectively. Despite their distinct geographic ranges, AHFV and KFDV share a remarkably high sequence identity. Given its emergence decades after KFDV, AHFV has since been considered a variant of KFDV and thought to have arisen from an introduction of KFDV to Saudi Arabia from India. To gain a better understanding of the evolutionary history of AHFV and KFDV, we analyzed the full length genomes of 16 AHFV and 3 KFDV isolates.

**Methodology/Principal Findings:**

Viral genomes were sequenced and compared to two AHFV sequences available in GenBank. Sequence analyses revealed higher genetic diversity within AHFVs isolated from ticks than human AHFV isolates. A Bayesian coalescent phylogenetic analysis demonstrated an ancient divergence of AHFV and KFDV of approximately 700 years ago.

**Conclusions/Significance:**

The high sequence diversity within tick populations and the presence of competent tick vectors in the surrounding regions, coupled with the recent identification of AHFV in Egypt, indicate possible viral range expansion or a larger geographic range than previously thought. The divergence of AHFV from KFDV nearly 700 years ago suggests other AHFV/KFDV-like viruses might exist in the regions between Saudi Arabia and India. Given the human morbidity and mortality associated with these viruses, these results emphasize the importance of more focused study of these significant public health threats.

## Author Summary

Alkhurma hemorrhagic fever (AHF) and Kyasanur Forest disease (KFD) viruses both cause serious and sometimes fatal human disease in their respective ranges, Saudi Arabia and India. AHFV was first identified in the mid-1990s and due to its strong genetic similarity to KFDV it has since been considered the result of a recent introduction of KFDV into Saudi Arabia. To gain a better understanding of the evolutionary history of AHFV and KFDV, we sequenced the full-length genomes of 3 KFDV and 16 AHFV. Sequence analyses show a greater genetic diversity within AHFV than previously thought, particularly within the tick population. The phylogeny constructed with these 19 full-length sequences and two AHFV sequences from GenBank indicates AHFV diverged from KFDV almost 700 years ago. Given the presence of competent tick vectors in the regions between and surrounding Saudi Arabia and India and the recent identification of AHFV in Egypt, these results suggest a broader geographic range of AHFV and KFDV, and raise the possibility of other AHFV/KFDV–like viruses circulating in these regions.

## Introduction

Alkhurma hemorrhagic fever virus (AHFV) is a variant of Kyasanur Forest disease virus (KFDV), and like KFDV, is a member of the mammalian tick-borne encephalitis group [family *Flaviviridae*, genus *Flavivirus*]. AHFV and KFDV cause similar disease syndromes in humans marked by fever, severe headache, myalgia and arthralgia, followed in a subset of cases by a hemorrhagic syndrome or diffuse neurological sequelae [1]–[4]. Recent epidemiological studies of AHFV outbreaks describe case fatality ratios close to those associated with KFDV infection (2–10%), suggesting that previous estimates of 25% might have been exaggerated due to unrecognized asymptomatic or mild cases [5]. Beyond supportive care, there is no specific treatment for either AHFV or KFDV infections.

**Figure 1:**
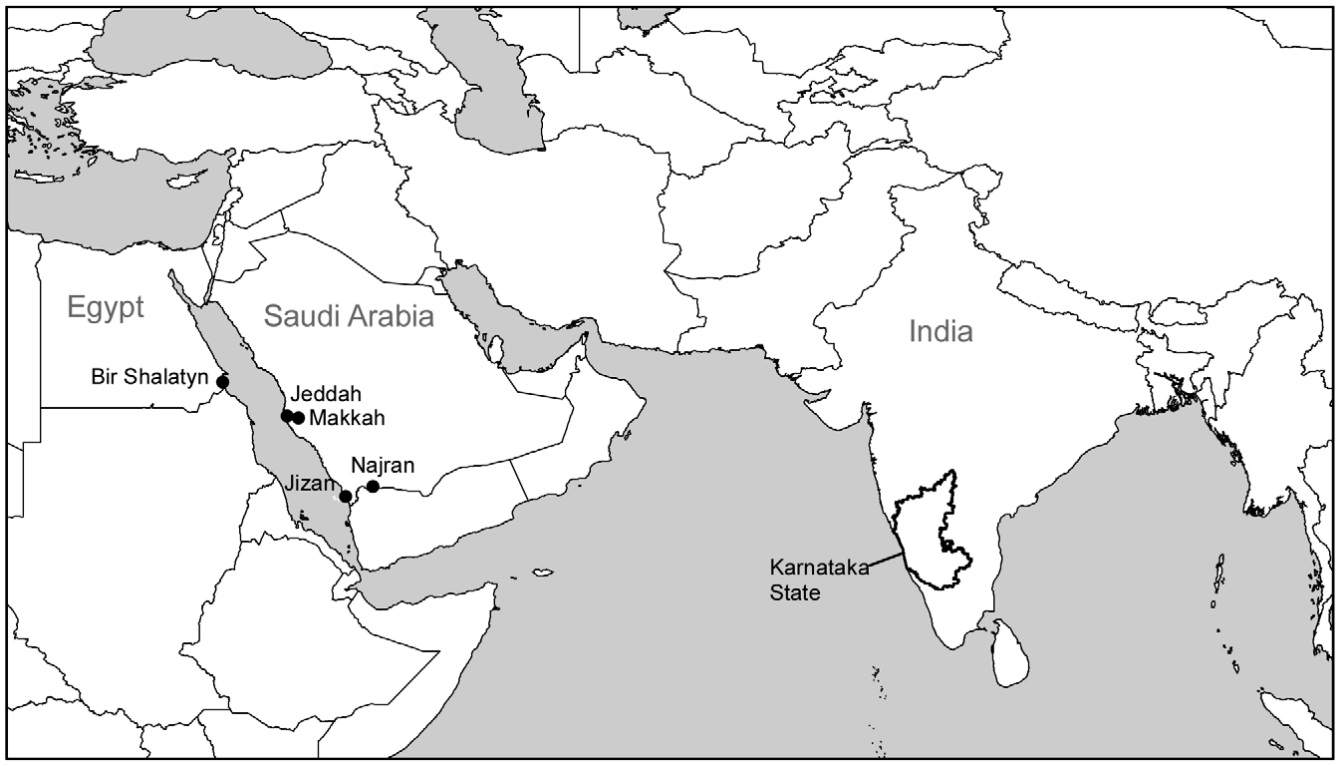
Map of regions where AHFV or KFDV have been isolated. AHFV cases have been documented in southern and western Saudi Arabia (Jizan/Najran and Jeddah/Makkah), and most recently at the Egypt-Sudan border (Bir Shalatyn or Shalatin). KFDV cases have been confined to Karnataka State, India (outlined).

AHFV and KFDV have distinct geographic ranges in Saudi Arabia and India, respectively (Figure 1). AHFV was isolated in 1994 in Makkah, Saudi Arabia, from the blood of a fatally infected butcher [6]. Since then, AHFV cases have been confirmed in Jeddah, Jizan, and Najran, and most recently, outside of Saudi Arabia near the Egypt-Sudan border in 2010 (Figure 1) [7]. Some early reports used an alternate spelling of Alkhumra [1], [2]. Human AHFV infections have been associated with tick bites, and AHFV has been isolated from an *Ornithodoros* tick in Jeddah [8], and *Ornithodoros savignyi* and *Hyalomma dromedarii* ticks in Najran [9]. However, another common risk factor for AHFV infection appears to be close contact with domestic animals, particularly sheep and camels [5], although no disease has been reported in livestock or other animals. The host range of KFDV is quite different from AHFV; KFDV was first identified as the cause of nonhuman primate die-offs (*Presbytis entellus* and *Macaca radiata*) as well as a concurrent outbreak of fatal human disease in the Kyasanur forest region of India in 1957. All known confirmed laboratory cases of KFD in people have been associated with contact with ticks (primarily *Haemaphysalis spp.),* activities within the Shimoga forest region, or through laboratory infections.

Despite apparent differences in their hosts and geographic ranges, AHFV and KFDV share high sequence identity [10], [11]. Their positive-sense RNA genomes are approximately 11 kb in length and encode a single 3416 amino acid polyprotein that is post-translationally cleaved into a total of 3 structural (C, M and E) and 7 nonstructural (NS1, NS2a,NS2b, NS3, NS4a, NS4b, and NS5) proteins. Given the notable genetic similarity and the later emergence of AHFV in Saudi Arabia, it has been speculated that AHFV arose following an introduction of KFDV from India. Previous phylogenetic analyses of AHFV [12] and KFDV [10] have relied on partial gene sequences, including regions of the structural envelope (E), the RNA-dependent RNA polymerase (NS5), and the viral protease/helicase (NS3). These studies indicated a recent divergence of AHFV and KFDV, occurring sometime between 1828 and 1942, presumably spawned by the introduction of KFDV into Saudi Arabia. However, given that the vector ecology, mammalian hosts and ecological niche of AHFV differ markedly from KFDV, it is possible that a longer period of divergent evolution between AHFV and KFDV could explain these significant biological differences.

A more complete understanding of the evolutionary history of these viruses would provide insight into the circumstances surrounding their emergence. This is particularly important, as AHFV and KFDV are serious public health threats, and the recent identification of AHFV in Egypt demonstrates the potential for either viral spread and range expansion, or a larger range than previously thought. To gain insight into the relationship between AHFV and KFDV, we sequenced the full-length genomes of 16 AHFV and 3 KFDV isolates and analyzed those with two existing sequences available in GenBank. Our analyses revealed a higher overall diversity amongst AHFV strains than previously thought, particularly within the tick population. Surprisingly, these analyses indicated a much earlier divergence of AHFV from KFDV approximately 700 years ago, suggesting AHFV and KFDV might have broader geographic ranges, and raises the possibility of closely related but undiscovered virus variants existing in the regions between Saudi Arabia and India.

## Methods

### Ethics statement

Animal procedures in this study complied with institutional guidelines, the US Department of Agriculture Animal Welfare Act, and the National Institutes of Health guidelines for the humane use of laboratory animals. Procedures were approved by the Centers for Disease Control and Prevention (CDC) Institutional Animal Care and Use Committee (IACUC).

### Virus isolates

The virus isolates included in this study are listed in Table 1. All AHFV samples were received from the Saudi Arabia Ministry of Health. Partial sequences of the AHFV tick pool isolates have been published earlier [9]; the human AHFV samples had not been previously reported. Viruses were isolated through inoculation of sucking mice. Animals showing clinical symptoms were euthanized and brain suspensions were prepared in Hanks balanced salt solution and clarified by low-speed centrifugation, and the supernatant was used to infect Vero E6 cells. The KFDV isolates had been cataloged by the National Institute of Virology (NIV) in Pune, India after isolation in 1957 [10]. These lyophilized isolates were reconstituted in RNase-free water and used to infect Vero E6 cells. The cells were incubated at 37°C for 5 days, and then supernatants were harvested for RNA extraction.

**Table 1:** AHFV and KFDV isolates included in analysis.

Isolate	Year isolated	Location	Source	Length (nt) sequenced	GenBank Accession
KFDV-P9605	1957	Karnataka	Human	10774	JF416958
KFDV-G1138	1957	Karnataka	Tick (*H. spinigera*)	10694	JF416959
KFDV-W377	1957	Karnataka	*S. entellus*	10694	JF416960
AHFV-809006	1994	Makkah	Human	10775	JF416957
AHFV-807321	1995	Makkah	Human	10749	JF416956
AHFV-1176*	1995	Makkah	Human	10685	AF331718
AHFV-200201032	2002	Makkah	Human	10749	JF416949
AHFV-200201034	2002	Makkah	Human	10749	JF416950
AHFV-200201035	2002	Makkah	Human	10749	JF416951
AHFV-200201036	2002	Makkah	Human	10749	JF416952
AHFV-200204519	2002	Najran	Human	10749	JF416953
AHFV-200300001	2002	Makkah	Human	10749	JF416954
AHFV-200307463	2003	Jizan	Human	10749	JF416955
AHFV-JE7*	2004	Jeddah	Tick (*O. savigyni*)	7367	DQ154114
AHFV-200904529	2009	Najran	Tick (*H. dromederii*)	10749	JF416961
AHFV-200905915	2009	Najran	Tick (*O. savigyni*)	10749	JF416963
AHFV-200905916	2009	Najran	Tick (*O. savigyni*)	10749	JF416964
AHFV-200905919	2009	Najran	Tick (*O. savigyni*)	10749	JF416965
AHFV-200905921	2009	Najran	Tick (*O. savigyni*)	10749	JF416966
AHFV-200905922	2009	Najran	Tick (*O. savigyni*)	10749	JF416967
AHFV-200905926	2009	Najran	Tick (*O. savigyni*)	10749	JF416962

AHFV JE-7 and AHFV 1176 sequences were taken from GenBank (Acc. # DQ154114.1 and AF331718, respectively). With those exceptions (AHFV JE-7 and AHFV 1176) all other sequences terminate at the viral 3′ terminus. Longer sequences of AHFV 809006 and KFDV P9605 are due to sequencing of the 3′ RACE products of the complement strand of viral RNA. Shorter sequences of KFDV G11338 and KFDV W-377 resulted from the use of a different 5′ forward primer (6F instead of 1F). SMB – suckling mouse brain; E6 – Vero E6 passage; M – mouse.

### RNA extraction

To extract the viral RNA, cellular supernatant was added at a 1∶10 ratio to Roche Tripure isolation reagent (Roche Applied Science). The Tripure mixture was transferred to new tubes and surface decontaminated for removal from a biosafety level (BSL)-4 laboratory to a BSL-3 facility (both laboratories are registered and approved by the National Select Agents program). 250 µL chloroform was added to the Tripure mixture, the tubes vortexed and centrifuged at 10,000 rpm for 15 min. The aqueous layer was added to an equal volume of 70% ethanol. The remaining procedure was carried out per manufacturer's instructions. RNA was eluted in 50 µL of RNase-free water.

### Reverse transcription polymerase chain reaction (RT-PCR)

Three primer pairs were used to produce overlapping products of approximately 4 kb in length (Table 2) using the Invitrogen Superscript III One-step RT-PCR with Platinum Taq DNA Polymerase kit (Invitrogen). Two isolates, KFDV W-377 and KFDV G11338, were amplified from the 5′ end with an alternate forward primer (6F). For each reaction, 5 µl RNA was added to 25 µl of the 2x Reaction mix, 10 µM of each primer and 1 µL of Superscript RT-PCR Taq mix in RNase-free water. The one-step RT-PCR protocol was as follows: reverse transcription at 52°C for 30 min, followed by denaturation at 94°C for 2 min and 40 cycles of 94°C for 15 s, 58°C for 30 s, 68°C for 4 min, and a final extension of 68°C for 5 min.

**Table 2:** Primers used for amplification of AHFV and KFDV.

Primer	Location	Sequence (5′ – 3′)
Alk1F	27-46	GTTTCAGACAACGTGAGTGG
Alk6F	81-100	GAAGCGTTAACGTGTTGAGG
Alk5R	3666-3648	CAGTTACGACTAGGCCAAG
Alk2F	3487-3505	AACAGGGTGGTCTGGTGAG
Alk2R	7074-7055	AGGGAGTGAACAGAGAGACG
Alk7F	6835-6852	GGAAACAGCGAAGTAGCG
alkG1R	10775-10755	AGCGGATGTTTTTTCCGAAAC
Oligo dT	N/A	Invitrogen 3′ RACE-AP
3′RACE (v)	9925-9943	CAGCATGTCTCTCAAAGGC
3′RACE (vc)	324-306	TCGCCCAGAATCGCTTGAG

All primers successfully amplified KFDV and AHFV isolates (with the exceptions of AHFV JE-7 and AHFV 1176 that were derived from GenBank); however, efficient amplification of KFDV G11338 and KFDV W-377 required use of primer 6F, resulting in a slightly shorter final sequence.

### 3′ RACE

To confirm the 5′ and 3′ ends of KFDV and AHFV, 3′ RACE was performed on the viral RNA (vRNA) and viral complementary RNA (vcRNA) of one KFDV isolate (KFDV P9605) and one AHFV isolate (AHFV 809006). A polyA tail was added to both vRNA and vcRNA using the A-Plus Tailing kit (Epicentre Biotechnologies) in 50 µL reactions containing 35 µL RNA, 5 µL 10× A buffer, 5 µL 10mM ATP, 0.5 µL RNase-out, 1 µL polyA polymerase, and RNase-free water. Reactions were incubated for 15 min at 37°C, cleaned using Qiagen RNeasy columns (Qiagen Inc.), and eluted into 50 µL of RNase free water. A 3′ RT-PCR on vRNA and a 3′ RT-PCR on vcRNA were run separately, each with the Invitrogen 3′RACE-AP primer and a gene-specific primer (Table 2). Superscript III RT-PCR with Platinum Taq (Invitrogen) was used in 50 µL reactions containing 5 µL RNA with polyA tail, 2 µL gene-specific primer (3′ or 5′), 2 µL oligo dT primer, 25 µL 2x buffer, 1 µL enzyme mix, and 15 µl RNase-free water. Reactions were run at 50°C for 30 min and 94°C for 2 min, and then through 35 3-step cycles of 94°C for 30 s, 56°C for 30 s, and 68°C for 1min, with a final extension of 68°C for 5 min. Results from the genomic 3′ RACE were used to design the terminal 3′ primer (alkG1R, Table 2).

### Complete genome sequencing

PCR amplified products were sequenced using ABI Big-Dye 3.1 chemistry with an ABI 3730XL sequencer (Applied Biosystems). All available AHFV and KFDV isolates in GenBank were aligned and a panel of 52 sequencing primers was designed to encompass the entire genome. The chromatogram data were assembled and analyzed as described previously [13]. Approximately 85-90 reads were obtained for each genome, resulting in an average six-fold redundancy at each base position.

### Sequence analysis

The 16 AHFV and 3 KFDV full-length sequences were compared to 2 available AHFV sequences in GenBank: the prototype AHFV strain 1176 (GenBank Acc. #AF331718) and the partial coding sequence of AHFV strain JE-7 isolated from a tick (GenBank Acc. #DQ154114), for a total of 18 AHFV and 3 KFDV. All sequence alignments and pairwise comparisons were completed with MacVector with Assembler 11.1.2 (MacVector, Inc.). Estimation of the gene-specific ratio of nonsynonymous to synonymous mutations were completed using DataMonkey webserver [14]–[16]. Using ModelTest [17], the General Time Reversible model with a gamma distribution (GTR+G) was selected as the appropriate nucleotide substitution model. Marginal likelihood analysis indicated a relaxed lognormal clock [18] and a constant population size model was appropriate for the dataset. A Bayesian coalescent phylogenetic analysis was used to determine rates of evolution and time to most recent common ancestor (TMRCA) using BEAST 1.4.7 and Tracer 1.4 [19]. For the final analysis, 40,000,000 generations were run to ensure effective sample sizes (ESS) greater than 200. TreeAnnotator and FigTree 2.0 were used to build and visualize trees [19]. After completion of these full length genome analyses, small partial sequence fragments of the E and NS5 from an AHFV recently isolated in Egypt were published (GenBank Acc. #HM629507, HM629508) [7]. To build an inclusive phylogeny containing this new data, the analyses described above were rerun separately to include these partial gene fragments.

## Results

Here we describe the first AHFV/KFDV phylogeny produced using full-length genomes. To sequence the available 16 AHFV and 3 KFDV isolates (Table 1), primer pairs were designed to amplify 3 overlapping segments of approximately 4 kb each. These RT-PCR products span the entire 10.7 kb genome excluding the terminal 26 nucleotides (nt) of the non-coding 5′ untranslated region (UTR). The terminal 33 nt of the 5′ UTR and the terminal 39 nt of the 3′ UTR of KFDV-P9605 and AHFV-809006 were identical using 3′ RACE on the vRNA and vcRNA. The full genome lengths of AHFV and KFDV differed by only 1 nt; a conserved A in position 10415 of the noncoding 3′ UTR of AHFV was absent in KFDV, resulting in genome sizes of 10775 and 10774, respectively.

Analysis of full-length AHFV and KFDV genomes (Table 3) confirmed a high level of sequence identity between the viruses, with an overall diversity of 8.4% (nt) and 3.0% (aa). The region showing the most nucleotide diversity (11.6%) within AHFV isolates was the 23 aa (69nt) C-terminus of the NS4A protein, referred to as the 2K peptide [20]. However, the 2K amino acid sequence was completely conserved amongst AHFV isolates. The ratios of nonsynonymous to synonymous substitutions were determined for each AHFV were determined and showed no strong indication of positive selection.

**Table 3:** Gene-specific genetic diversity.

Gene	AHFV (dN/dS)	KFDV	AHFV v. KFDV	AHFV v. OHFV
C	2.1 (0.33)	0.0	6.9	30.1
prM	1.7 (0.24)	0.0	8.6	32.2
M	2.7 (0.25)	0.0	10.2	41.0
E	1.1 (0.11)	0.3	8.3	29.1
NS1	1.4 (0.14)	0.3	8.9	30.8
NS2a	1.5 (0.17)	0.3	8.7	37.7
NS2b	1.5 (0.10)	0.0	6.2	29.4
NS3	1.1 (0.11)	0.2	7.1	27.7
NS4a	1.6 (0.11)	0.0	8.7	36.6
NS4b	1.3 (0.24)	0.1	7.4	32.1
2K	2.9 (0.00)	0.0	11.6	28.1
NS5	1.0 (0.16)	0.2	7.2	24.7
Diversity (nt)	1.1	0.2	8.4	29.4
Diversity (aa)	0.8	0.1	3.0	22.2

Maximum pairwise (nt) comparisons (listed as percentages) were made within AHFV sequences (2^rd^ column), within KFDV sequences (3^rd^ column), and between AHFV and KFDV sequences (4^nd^ column). The 5^th^ column shows maximum pairwise (nt) comparisons between AHFV and Omsk hemorrhagic fever virus (OHFV), another tick-borne hemorrhagic flavivirus (GenBank Acc. AY438626.1). The ratio of nonsynonymous to synonymous mutations (dN/dS) for AHFV are included in the 2^nd^ column (in parentheses).

**Figure 2:**
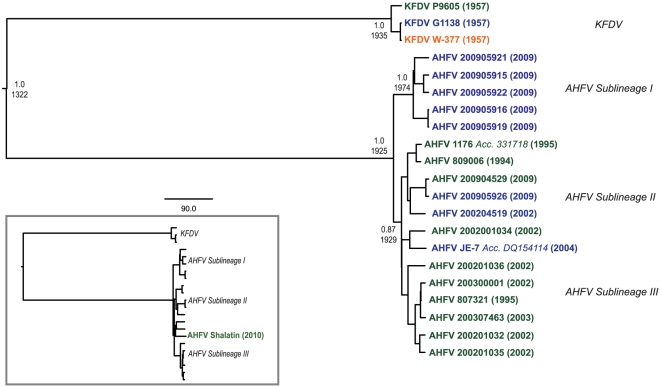
AHFV and KFDV full-length genome phylogeny. Phylogeny is based on Bayesian coalescent analysis of AHFV and KFDV full-length sequences. For selected clades, posterior probability values are listed above the TMRCA. Text color indicates host species: human isolates (green), tick pool isolates (blue), and nonhuman primate isolate (orange). The GenBank accession numbers are JF416949-67. Inset: Full-genome phylogeny with inclusion of short sequence fragments from 2010 Egyptian isolate (Shalatin, GenBank Acc. #HM629507, HM629508) shown in green.

The mean rate of molecular evolution was estimated to be 9.2×10^−5^ sub/site/year (95% highest posterior density [HPD]: 1.6×10^−5^ sub/site/year [low] to 1.9×10^−4^ sub/site/year [high]). The phylogeny clearly showed AHFV and KFDV as 2 separate lineages (Figure 2). The TMRCA for all AHFV isolates was recent, 84 years before 2009 (∼1925) [HPD range: 29-165 years]. All KFDV isolates shared a recent TMRCA as well, 75 years before 2009 (∼1934) [HPD range: 56–104]. Despite their genetic similarity and individual recent TMRCAs, our analyses revealed that AHFV and KFDV diverged approximately 687 years before 2009 [HPD range: 121-1487 years].

This analysis encompassed 18 AHFVs isolated from either southern or western Saudi Arabia. Eight AHFVs were derived from ticks, including one published previously [8]. The remaining 10 were derived from human cases, including the prototypic AHFV 1176, also previously published [11]. The resulting phylogeny showed all known AHFV isolates fall into 3 sublineages (Figure 2). Sublineage I consisted entirely of 5 tick pool isolates from the Al Balad Magan camel market in Najran. All were isolated from *Ornithodoros savigyni* ticks and had a pairwise diversity of 0.5%. Sublineage II included two tick pool isolates taken at the same time from a nearby camel market, also in Najran, in the Al Mishaaliyia district. Despite the close proximity of the camel markets, the genetic variation between tick pool isolates of Sublineage I and II was 1.1%, slightly higher than the overall diversity seen within all human AHFV isolates (0.7%). The tick pool isolates of Sublineage II came from different tick species (one from *Hyalomma dromedarii* and one from *O. savignyi*) but differed from one another by only 3 nt. There were no defining amino acid changes found between human and tick isolates. Sublineage II includes isolates from 1994-2009, the full range of available full-length genomes, as well as the small gene fragments from the 2010 Egyptian isolate. Sublineage III encompasses isolates taken from ticks and humans in Makkah, Jeddah and Jizan. In summary, the genetic differences between AHFV isolates did not obviously correlate with geographic, temporal or host origin.

## Discussion

The emergence of AHFV in the mid-1990s and its remarkable genetic similarity to KFDV suggested that AHFV arose from a recent introduction of KFDV from India into Saudi Arabia. Using partial E and NS5 sequences of KFDV, the AHFV/KFDV divergence was estimated to have occurred 68 years ago [10]. Similarly, Charrel et al., found a TMRCA of 182 years ago with partial E, NS3, and NS5 sequences of AHFV [12]. Using only the concatenated E and NS5 sequences of our isolates (as in [10]), we also found a recent time of divergence (∼100 years before 2009). However, analyzing full-length genome sequences, we show 2 distinct lineages, with KFDV and AHFV diverging approximately 687 years ago. Analyzing full-length genomes allowed us to incorporate the full diversity of AHFV isolates, and therefore to provide a more accurate estimate of an older common TMRCA.

The use of full genomes also revealed a slower rate of molecular evolution (9.2×10^−5^ substitutions/site/year) than determined previously with partial sequences of KFDV [10]. This may be explained by the slightly higher NS5 and E diversity in KFDV relative to the rest of the genome (Table 3), however, the limited number of KFDV isolates available for this study might not be fully representative of the extant KFDV diversity. In accordance with the evolutionary rate patterns for vector-borne flaviviruses [21], the AHFV/KFDV rate is slower than mosquito-borne flaviviruses, including Dengue virus (DENV) [22] and yellow fever virus (YFV) [23] and more similar to tick-borne flaviviruses, including the closely related tick-borne encephalitis virus (TBEV Siberian subtype, 1.64×10^−4^ sub/site/year) [24]. The evolutionary rate is also similar to that of an unrelated tick-borne member of the *Bunyaviridae* family, Crimean-Congo hemorrhagic fever virus (0.58–1.52×10^−4^ sub/site/year)[25].

In our analyses, the sublineages of AHFV isolates did not show clustering by geography or date of isolation. Although these isolates primarily came from two distinct geographic areas, the Jeddah/Makkah region in the west and the Jizan/Najran region in the south, frequent travel of people and livestock between these regions effectively eliminates geographic boundaries. The short sequences available from the 2010 Egyptian isolate placed it within Sublineage II, the most inclusive of AHFV sublineages, possibly suggesting a relatively recent introduction to Egypt from Saudi Arabia. Alternatively, the limited sequence data available from the Egyptian isolate is not representative of extant diversity within East Africa and the full-length sequence could indicate a different evolutionary history. The narrow range of time encompassed by the viral isolates (∼15 years) and the slow evolutionary rate of AHFV may also explain the lack of detectable temporal clustering.

The centuries between the divergence of the ancestral AHFV and KFDV (∼year 1322), and the individual TMRCAs of AHFV (∼1925) and KFDV (∼1933) isolates, coupled with a slow evolutionary rate, indicate the 2 viruses evolved separately and AHFV is not a result of recent KFDV introduction in Saudi Arabia [10]. This separate evolutionary history spanning several hundred years and the vast geographic distance involved raises the possibility of an existing spectrum of thus far unknown tick-borne encephalitic/hemorrhagic viruses in the regions between Saudi Arabia and India. In support of such a viral spectrum in that region, Gould et al. [26] cite the presence of close AHFV/KFDV relatives, Karshi virus (KSIV) and Farm Royal virus (FRV), in Uzbekistan and Afghanistan, respectively. A similar dispersal corridor of TBEV has been described from Eastern Europe westwards [27], although more recent studies did not support such a pattern [28]. Dispersal of the ancestral viruses of AHFV and KFDV may have been accomplished through the movement of animals, including camels presumably carrying ticks, along the Silk Road, which by the 1300s stretched from Europe to China.

The presence of potentially competent tick vectors in these regions [29] also supports this possibility of AHFV/KFDV-like viruses existing between Saudi Arabia and India. Although the viruses are primarily associated with two tick genera, AHFV with *Ornithodoros* and KFDV with *Haemaphysalis*, both have been isolated from multiple genera. At least 16 tick species have been shown to transmit KFDV [3]. The experimental finding of transovarial and transtadial transmission of KFDV in *O. cruzi* [30], coupled with the ubiquitous nature of *Ornithodoros spp.* in India, Saudi Arabia and the intermediate regions, suggests these ticks may have played an important role in the spread of AHFV/KFDV-like viruses. Although the transmission of KFDV has been primarily associated with *H. spinigera,* a tick species not documented in Saudi Arabia, these ticks are present between Saudi Arabia and India [29]. Given the diversity of potential vectors and their extensive ranges, it is feasible that AHFV and KFDV, as well as other similar but thus far undiscovered viruses, are circulating more broadly throughout Saudi Arabia, India and beyond.

The genomic diversity of AHFV was most apparent within isolates from the tick population. Ticks sampled on the same day in one city (Najran) harbor a more genetically diverse AHFV population than isolates obtained from human cases over the course of 15 years throughout Saudi Arabia. Similarly, an analysis of the European subtype of tick-borne encephalitis virus (TBEV) isolated from ticks shows relatively high variation in a limited geographic area and temporal period, perhaps due to importation of TBEV on migratory birds [31], [ 32]. The notable genetic heterogeneity of AHFV in Najran may be explained similarly, as camel and other livestock traders often travel large distances to Najran to market their animals. Like all soft ticks, *Ornithodoros* spp. feed for only short periods, spending most of their time in burrows or nests, allowing areas such as a camel market to become a potential focus of AHFV infection and high virus genomic diversity. Presumably, like KFDV, AHFV can persist for long periods within a tick, and given the nature of RNA viruses, variants may evolve over time within the invertebrate hosts. The lower diversity within human cases may reflect limited transmissibility or reduced pathogenicity of some of these variants in people.

In our analysis, the use of full-length AHFV and KFDV genomes demonstrated a deeper evolutionary history than suggested by previous partial genome analyses. The divergence of AHFV and KFDV almost 700 years ago indicates a long period of divergent evolution, and suggests that a range of as-yet undiscovered tick-borne hemorrhagic/encephalitic viruses could exist between Saudi Arabia and India. The notably high AHFV diversity found within tick populations, coupled with the extensive geographic range of competent tick vectors, raises the possibility of broader AHFV and KFDV geographic ranges, and is supported by the recent discovery of AHFV in Egypt. As AHFV and KFDV are both associated with significant human morbidity and mortality, the potential spread of these viruses should be of serious concern and warrants further study of these significant pathogens.
